# The Significance of Occupants’ Interaction with Their Environment on Reducing Cooling Loads and Dermatological Distresses in East Mediterranean Climates

**DOI:** 10.3390/ijerph18168870

**Published:** 2021-08-23

**Authors:** Jihan Muhaidat, Aiman Albatayneh, Mohammed N. Assaf, Adel Juaidi, Ramez Abdallah, Francisco Manzano-Agugliaro

**Affiliations:** 1 Department of Dermatology, Faculty of Medicine, Jordan University of Science and Technology, P.O. Box 3030, Irbid 22110, Jordan; jmmuhaidat@just.edu.jo; 2 School of Natural Resources Engineering and Management, German Jordanian University, P.O. Box 35247, Amman 11180, Jordan; aiman.albatayneh@gju.edu.jo (A.A.); M.assaf@gju.edu.jo (M.N.A.); 3 Mechanical and Mechatronics Engineering Department, Faculty of Engineering and Information Technology, An-Najah National University, P.O. Box 7, Nablus 00970, Palestine; ramezkhaldi@najah.edu; 4 Department of Engineering, ceiA3, University of Almeria, 04120 Almeria, Spain

**Keywords:** occupant’s behavior, cooling load, East Mediterranean, Jordan, heat health distresses, sustainability, dermatological diseases

## Abstract

Global endeavors to respond to the problems caused by climate change and are leading to higher temperatures inside homes, which can cause skin conditions (such as eczema), lethargy, and poor concentration; disturbed sleep and fatigue are also rising. The energy performance of buildings is influenced by interactions and associations of numerous different variables, such as the envelope specifications as well as the design, technologies, apparatuses, and occupant behaviours. This paper introduces simple and sustainable strategies that are not dependent on expensive or sophisticated technologies, as they rely only on the actions practiced by the building’s occupants (movable window shading, and nighttime natural ventilation) instead of completely relying on high-cost mechanical cooling systems in buildings located in the main Eastern Mediterranean climates represented in the country of Jordan. These low-energy solutions could be applied to low-income houses in hot areas to avoid health problems, such as dermatological diseases, and save a significant amount of energy. The final results indicate that window shading has significant potential in reducing the cooling load in different climate zones. Natural ventilation exhibits high energy-saving abilities in climates that have cool nights, whereas its abilities in hot climates where nights are moderate is limited.

## 1. Introduction

The building sector is considered one of the most energy-intensive sectors worldwide. The International Energy Agency (IEA) indicates that in the majority of its member countries, 40% of raw energy is consumed by the building sector [1]. In Jordan, the building sector accounts for 21.5% and 45.4% of the final energy and total electrical usage, respectively. Therefore, improving the energy performance of buildings has become a high priority since it comprises a substantial share of the final energy consumption.

Occupant behaviour is classified as a primary factor that has a significant impact on building energy performance. The way in which occupants deal with energy can have a considerable influence on the energy consumption rate in the building [2,3,4,5]. Some research has attributed the variation between predicted energy and actual energy use to how occupants approach the issue of energy use [6,7,8,9]. Considering occupant behaviour as a stochastic rather than a deterministic system allows it to be controlled and therefore exploited effectively [9].

Several scientific works have indicated that climate change has led to a significant increase in the mean temperature range, which is reflected in the augmentation of the cooling demand in the building, especially among residential buildings [10,11,12,13,14]. This perception is accumulated in the urban area due to the urban heat island phenomenon.

As a result of global warming caused by climate change, many populations around the world are being increasingly exposed to heat. On a global basis, it is witnessed that extreme temperatures are becoming increasingly common, with such events lasting longer and covering larger areas. From 2000 to 2016, approximately 125 million people around the world were impacted by heat waves. The effects that such elevated temperatures have on human health are dependent on how intense the heat is, how long it lasts, the ability of people to adapt, and whether buildings are suitably designed for the conditions. The way in which people react to heat depends on individual adaptation capabilities, and it has the ability to cause sudden effects. For this reason, building design is a critical factor, as it can reduce the impacts of increased temperatures on residents while minimizing energy for the purpose of maintaining thermal comfort [15].

### 1.1. Thermal Comfort and Human Health

Elevated temperatures caused by heat waves, with durations lasting for multiple days, can have a considerable effect on society, which can include an increase in deaths associated with heat. Although hot weather is a natural event that is considered particularly risky, it is not the focus of sufficient attention as mortality rates and the damage caused are not always clear. Between 1998 and 2017, an excess of 166,000 individuals lost their lives as a result of heat waves [16], and a particular heat wave that impacted Europe in 2003 caused the deaths of greater than 70,000 people [17]. Although the impact of heat is generally worsened in cities as a result of the heat island effect (i.e., urban regions have considerably higher temperatures compared to rural regions because of human activities. The temperature gap is generally wider at nighttime compared to daytime and is most obvious when winds are minimal), communities outside of urban areas can also experience significant disruptions to their health and livelihoods both during and after intense heat waves due to the pressure applied to energy, water, and transportation systems, which can lead to limited power or even complete blackouts.

Heat exposure has the potential to cause serious health conditions, including heat exhaustion or heat stroke (which can cause victims to faint), and can also result in warm, dry skin as the body has a limited ability to control elevated temperatures. Additional symptoms that can occur are lower limb swelling, heat rash around the neck, cramps, headaches, fatigue, irritability, and lethargy. Heat has the capability to make people severely dehydrated, can trigger acute cerebrovascular episodes, and is a contributory factor to thrombogenesis (blood clots). Individuals with chronic conditions who take drugs on a daily basis are at an increased risk of experiencing complications or death when heat waves occur, in addition to the elderly and those who are very young [15]. For example, the heat wave that recently impacted the Pacific Northwest resulted in the deaths of hundreds of people [18].

It has recently been reported that increased temperatures during the night that affect the ability to rest cause numerous old and infirm people to die. It is unfortunate that as a result of the urban heat island effect, temperatures at night are the most affected, which is caused by buildings and roads re-releasing heat that they have absorbed during the day.

Approximately 90% of the heat dissipated by the body is through the skin. When overheating occurs, more blood is pumped around the body; the dilation of blood vessels facilitates the greater blood flow, and the groups of small capillaries that thread the skin’s upper layers begin to function. The circulation of the blood around the body increases close to the surface of the skin, while surplus heat is released into the colder environment. Simultaneously, perspiration occurs as a result of the diffusion of water through the skin. In isolation, sweating does not contribute to cooling the body. The cooling of the body instead requires evaporation in order to remove the water, although evaporation is retarded by high levels of relative humidity. Essentially, evaporation cools the body because of the heat energy needed for extracting sweat from the body [19].

In general, heat disorders are caused when the ability of the body to release heat via circulatory changes and sweating is either reduced or collapses, or when a chemical (salt) imbalance occurs due to excessive sweating. In situations where the amount of heat gained is greater than the amount the body is capable of removing, or when the body is incapable of compensating for the loss of salt and fluids caused by sweating, the core temperature starts to increase, thus leading to heat-related disorders. Increased temperatures can also cause people to become dehydrated, experience heat exhaustion, or even heatstroke, which can be fatal. Intense heat waves can also worsen existing illnesses, including diabetes, respiratory disorders, kidney disease, and heart disease. Those living in cities, old people, young people, outdoor laborers, and individuals whose health is impaired and have restricted mobility are specifically vulnerable to heat-related disorders and death. As cutaneous infections are linked with higher temperatures, regions with such climates report higher rates of these types of infections, such as [20]:

- Bacterial infections, such as cellulitis, boils, and impetigo;
- Skin disorders caused by fungal infections, such as tinea pedis and pityriasis;
- Viral skin disorders; inflammatory skin disorders; the frequency and severity of contact dermatitis rise in line with an increase in the ambient temperature;
- Intertrigo is more common in higher temperatures, particularly when related to diabetes and obesity;
- Hyperhidrosis causes miliaria and transient acantholytic dermatosis (Grover disease);
- Increased temperatures can also lead to flares of rosacea, cholinergic urticaria, and heat urticaria.

### 1.2. Energy Saving through Solar Shading and Occupant’s Behaviour

Building occupants are critical in maintaining thermal comfort levels, as they can employ ventilation at night for cooling purposes, lower the heat load within the house, ensure that windows and shutters are closed (or install shades, curtains, awnings, or louvres on windows that are exposed to the sun in the morning or afternoon)—particularly for windows that face the sun in the daytime. Additionally, wet towels can be hung for cooling the air within rooms, but this can simultaneously cause an increase in the humidity of the air. Electric fans can also facilitate comfort, but only when temperatures dip below 35 °C. Furthermore, residents can take baths or showers for cooling purposes (cold wraps/packs, towels, sponging, foot baths), wear clothing made of natural materials that are light and breathable, frequently consume water, and eat smaller meals on a regular basis.

Solar shading machines are considered essential bioclimatic elements and are common techniques utilized by designers to enhance facade performance. Using solar shading devices can significantly enhance the energy efficiency in buildings by preventing unwanted solar heat during summer [21,22,23]. Therefore, several energy efficiency codes and guidelines recommend the use of solar shading techniques mainly to decrease solar heat gain in the summer season.

Moveable, vertical solar shading tools provide the ability to balance different indoor environmental quality features, each discomfort glare, view, privacy, and thermal comfort. Thus, they are broadly employed in buildings and have proven to be effective in reducing overheating during summer [24,25]. Simulation work has shown that using movable window shading can lead to a decrease of around 14% in peak cooling and result in savings of 9.8% in terms of the annual energy consumption in tropical climates [26]. Another experimental study targeted at enhancing the energy performance of a primary school was conducted in Empoli, Italy, a region characterized by a Mediterranean hot summer climate. The results showed that controlling solar gain through the use of shading devices caused a 10–15% decrease in the cooling demand [27]. Additionally, the effect of utilizing shading in a residential building was investigated in a region with a hot summer climate in China. The outcomes demonstrated that besides the ability to decrease the energy requirement by 31%, movable shading further upgraded indoor thermal comfort by 21% during the summer [28].

Natural ventilation is not only important for achieving a comfortable and healthy indoor environment, but also for saving energy. Since ventilation allows external air to enter and take the place of internal air, it should be carefully controlled to exploit it properly. Thus, in terms of passive cooling, natural ventilation is of great importance [29,30,31]. In terms of natural ventilation strategies, nighttime ventilation is the most efficient as a result of its ability to reduce the energy consumption required for cooling spaces compared with daytime and full-day ventilation [32]. The night ventilation mechanism relies on a flow of cold nocturnal air that replaces the internal air that carries the heat gained during the day. Consequently, colder night air will cool the building structure and the interior air and will delay the rising of the indoor temperature during the day [33,34,35]. Resultantly, significant savings in cooling demand can be achieved when applying night ventilation [36,37,38,39,40,41].

Occupant behaviour and adaptation to the current environment can save a significant amount of cooling energy through the application of shading devices, the use of night ventilation, and the employment of adaptation techniques (wearing breathable clothes, drinking water, and using low-energy solutions, such as fans) instead of completely relying on mechanical systems. The amount of possible savings can exceed 50% of the cooling energy depending on the adaptation level and the awareness of the occupants [41,42,43].

Despite the significant role that occupant behaviour plays in buildings’ energy performance and the high potential for energy savings that can be achieved through the application of simple procedures, this area of research has received insufficient attention among different building stakeholders (designers, users, code developers, etc.), particularly in developing countries, such as Jordan. This paper aims to investigate the efficiency of applying smart and simple strategies (i.e., movable window shading and night-time natural ventilation) to reduce the cooling demand in residential buildings in different climates. Additionally, this research aims to highlight the importance of low-energy solutions, such as passive design strategies, for low-income houses in hot areas to avoid health problems, such as dermatological diseases. Based on the authors’ knowledge, this area of research has not been discussed before.

## 2. Methodology

In this section, for different Jordanian climate zones (these climates also represent the main climates in the South and East Mediterranean climate zones), the DesignBuilder simulation tool is used to examine the effect of occupants’ interaction with their environment on reducing cooling loads as well as the resulting effect this has on preventing various dermatological diseases.

### 2.1. Jordanian Climate Zones

**Figure 1:**
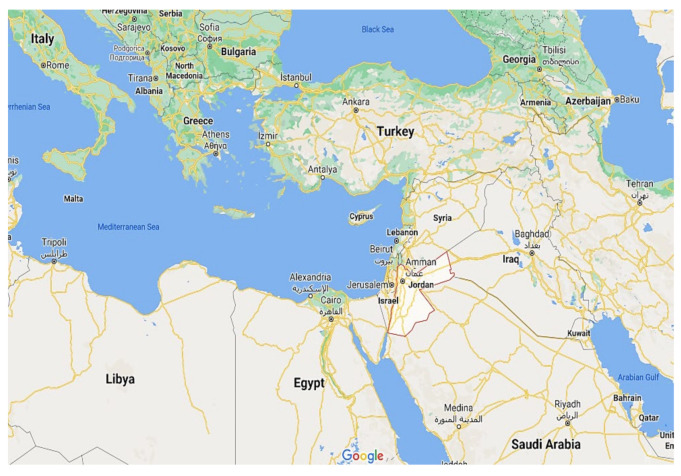
Jordan Location [44].

Jordan is located between the Arabian Desert and the Eastern Mediterranean area (see Figure 1), which explains the climatic conditions of the country that are characterised by long, hot, and dry summers and short, cold winters. In fact, December, January, and February are the coldest months, with maximum/minimum average temperatures equivalent to 10 °C to 5 °C, respectively. However, July, August, and September are considered to be the hottest months, with maximum/minimum average temperatures ranging from 35 °C to 20 °C. During the summer, the daily temperature increases considerably until reaching or exceeding 40 °C in some cases, particularly when a hot, dry, south-easterly wind blows. Generally, in winter, significant amounts of precipitation fall (between 200–400 mm) but decrease/stop during the summer season.

**Figure 2:**
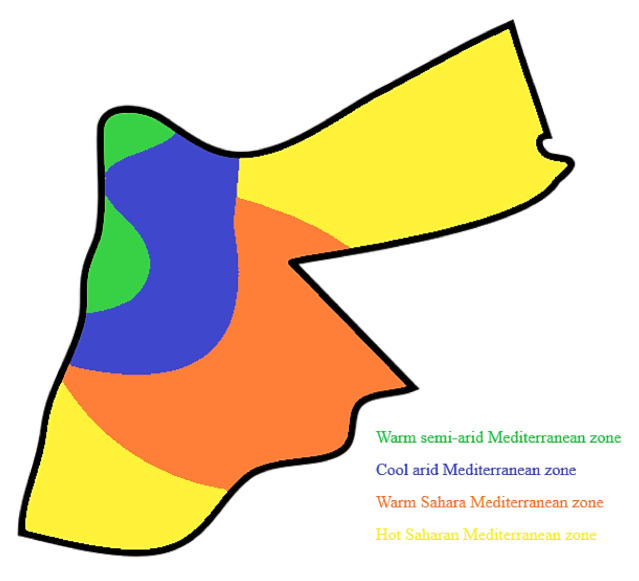
Jordanian main climates zones.

There are different climate zones in Jordan, some of which are similar to the Mediterranean climate and others that are almost like the desert climate. The Jordanian weather is characterised by four distinct seasons, as shown in Figure 2, where autumn and spring offer an ideal range for human comfort. The main climate zones in the major Jordanian cities are as follows [45]:

The warm, semi-arid Mediterranean zone is comprised of the major cities in Jordan (Amman, Irbid, Jerash, Madaba, Kerak), which have mild to warm summers and cold winters. The summer season is hot and dry with cool evenings, while the winter is cold, with an average temperature of 8 °C. Rainfall predominantly occurs between November and March, with the average annual rainfall reaching 300 mm and snow sometimes occurring. In general, spring and autumn are short and last around one month (spring in April and autumn in October).

The cool, arid Mediterranean zone is characterized by hot summers with a high sun glare and cold winters with significant temperature differences between day and night. The annual mean precipitation ranges between 100–150 mm, with a maximum temperature of 30–34 °C in the summer and a minimum temperature of 2–4 °C in the winter.

The warm Saharan Mediterranean zone’s main characteristics are hot to very hot dry summers with high sun glare during summer days. The daily hours of sunshine peak in August at 12 h, with an average temperature of 33 °C. Winter days may be warm during the day but cold during the night, with significant temperature differences occurring between day and night. The coldest temperature, of around 14 °C, is mainly observed in January, and the wettest month is December, with an average of 2.6 mm of rain.

The hot Saharan Mediterranean zone climates are characterized by year-round sunshine, stable air, and high pressure. The hot Saharan regions are generally warm and dry during the whole year. The summer is hot to extremely hot, with average temperatures reaching 35 °C, and during heat waves, the peak can reach 40 °C. During the colder days of the winter, temperatures can fall to freezing during the night.

### 2.2. Simulation Tool (DesignBuilder)

Many different simulation tools can be used for building simulation, and the accuracy level can vary depending on the tools themselves, the simulation data, and the actual data recorded in the real situation [46,47,48]. The energy performance of the case study building was examined using DesignBuilder software. DesignBuilder is an EnergyPlus-based software tool used to analyze building energy, including heating, cooling, lighting, and ventilation.

**Figure 3:**
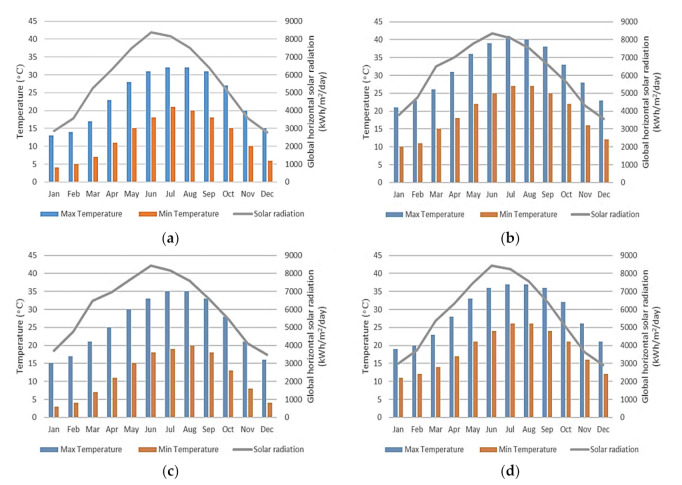
Average temperature and radiation profile for each climate. (**a**) Semi-arid Mediterranean, Warm. (**b**) Saharan Mediterranean, hot. (**c**) Saharan Mediterranean, warm. (**d**) Arid Mediterranean, cool.

The simulated model was used to analyze four Jordanian climates using hourly weather data, which is built into the DesignBuilder software. The relevant parameters included in the model simulation are: air temperature, dew point temperature, relative humidity, atmospheric pressure, global horizontal solar radiation, diffuse horizontal solar radiation, direct normal radiation, wind speed, wind direction, and cloud cover. For instance, the average temperature and radiation profile for each climate is shown in Figure 3.

This research aimed to highlight the importance of energy conservation and energy efficiency concepts. An additional aim was to optimize a building into a low-energy or zero-energy consumption building by performing energy-mapping, as well as analyzing the energy consumption and optimum design for the building, through the use of DesignBuilder software. Recently, several simulation technologies have been used to evaluate early design decisions by using simulation tools and software to quickly model and test alternatives.

DesignBuilder is an advanced simulation tool that uses an EnergyPlus dynamic simulation engine to examine and thermally analyze building performance under different weather conditions, from the concept stage until the designs stage, to examine the best energy-saving, passive design techniques. In this study, DesignBuilder was used to build a typical Jordanian building using local construction materials and different weather data at hourly time steps for the main climate zones in Jordan.

### 2.3. Case Study on Jordanian Building

The main climates of Jordan were investigated, starting with the warm, semi-arid Mediterranean climate, which has a moderate summer with a lower cooling load compared with other climates. The second one is the hot Saharan Mediterranean climate, which has extremely hot summers with heavy cooling consumption demands. The third zone is the cool, arid Mediterranean climate, and the fourth one is the warm Saharan Mediterranean climate, both of which experience significant demand for cooling. The cooling load was calculated for each climate zone, and the annual energy saving of the two proposed techniques was measured.

**Figure 4:**
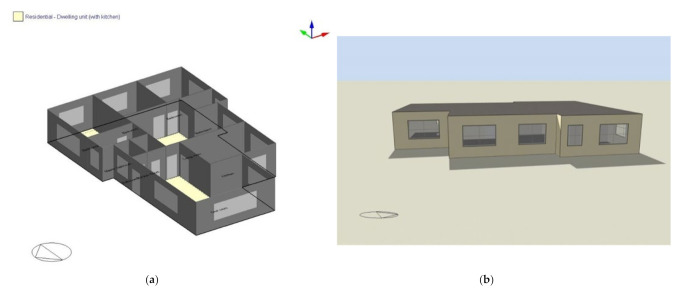
(**a**)**.** Top view and 3-D of the building in DesignBuilder. (**b**) Building overview.

A typical Jordanian house that adheres to the Jordanian building codes was used as the case study for this work. The model simulation building is built according to the current types of materials, building component configurations, structural designs, and construction details for the considered regions. This model is considered a baseline model for evaluating the performance of the proposed passive design strategies on the cooling demand. The simulation model is a residential building with an area of 186 m^2^, where the long axis faces the south and the building does not experience external shading or wind hurdles (Figure 4). The simulated model presented the best building features based on the Jordanian code; the aim of using such a model is to investigate the performance of smart and simple strategies (movable window shading and nighttime natural ventilation) in the high characteristics building; therefore, the other buildings (that do not follow the Jordanian code) are expected to experience larger reductions in the cooling demand using those strategies. The research output is presented in kWh/m^2^, which allows for easier extraction to other buildings.

The case study model was designed to follow the Jordan National Building Codes requirements for residential construction [49,50]. Table 1 explains the building features.

The shading was scheduled to be from 8 a.m. to 7 p.m. during the summer days, with sunrise occurring around 6:30 a.m. and sunset at approximately 7:30 p.m. Natural ventilation was scheduled from 7 p.m. to 8 a.m. The energy performance levels of scheduling window shading and natural infiltration were first examined individually and then in combination. The properties of the shading device are presented in Table 2.

## 3. Results and Discussions

**Figure 5:**
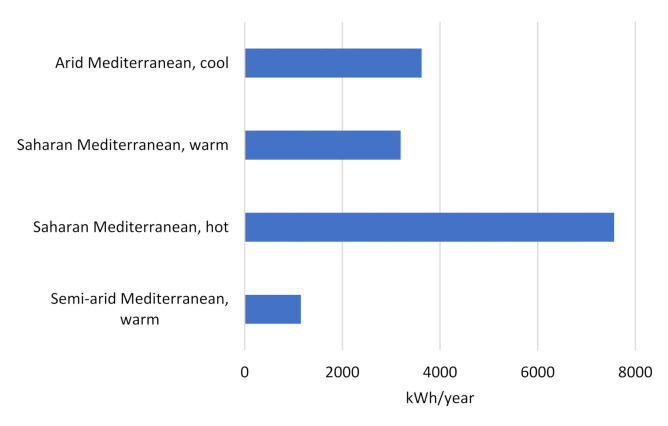
Cooling Consumption in Different Jordanian Climates.

**Figure 6:**
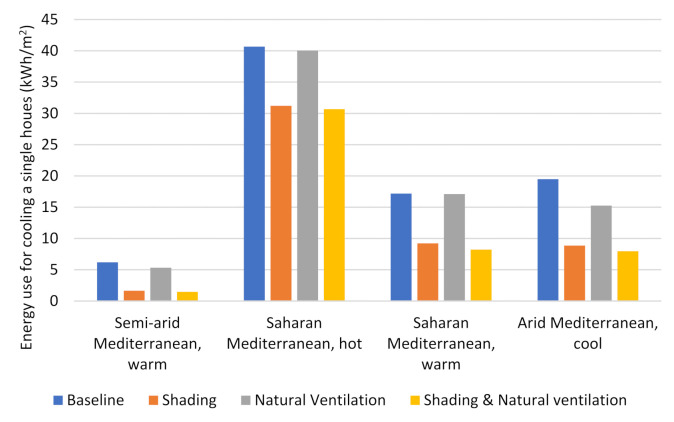
Annual energy consumption for residential buildings’ space cooling per area unit in different climates.

The cooling consumption was calculated for our case study building under different Jordanian climates, and a difference was observed in their relevant cooling demands. The highest cooling demand was found in the hot Saharan Mediterranean zone, while the most minimal demand was observed in the warm, semi-arid Mediterranean zone. The cool, arid Mediterranean and the warm Saharan Mediterranean zones show largely similar cooling demands, which are located between those for the highest and lowest zones (Figure 5 and Figure 6).

Table 3 shows the energy savings for cooling obtained via scheduled external window shading as well as the percentages for those savings relative to the total cooling demand for each climate. The highest energy savings were found in the cool, arid Mediterranean and warm Saharan Mediterranean climates, which were calculated to be 1910 and 1546 kWh/year, respectively. Those energy savings represent 52.69% and 48.4% of the cooling energy demand in the cool, arid Mediterranean and warm Saharan Mediterranean, respectively.

The energy-saving percentage reduced to around 23% in the hot Saharan Mediterranean, where the hottest climate conditions can be found. This could be explained by the fact that solar radiation is not the only climate factor that impacts the amount of cooling in hot regions. In these types of regions, other factors, such as ambient temperature, should be taken into consideration. Further, in hot climates, direct radiation does not only enter the building through window openings; the building can gain a significant amount of heat through conduction through other building envelope elements (wall, roof, etc.). The energy-saving results for the semi-arid Mediterranean climate amount to around 820 kWh/year, which accounts for 71% of the cooling consumption. Therefore, it can be considered that solar gain through windows constitutes the main source of heat transfer to the building in this climate.

Table 3 represents the amount of energy savings that can be made from cooling and the percentage of savings in the total amount of energy consumed for cooling when using nighttime natural ventilation. A significant amount of energy savings can be achieved in the arid Mediterranean and warm Saharan Mediterranean climates, reaching 444 (12.25%) and 356 (11.15%) kWh/year, respectively. This amount was reduced in the hot Saharan Mediterranean (119 kWh/year, 1.27%) and the warm, semi-arid Mediterranean (158 kWh/year, 13.74%). The difference in the energy-saving amounts between different climates is based on the temperature lag between day and night in addition to its dependence on the temperature dropping at night. Therefore, in hot climates that have warm nights, such as the hot Saharan Mediterranean, the natural ventilation technique has not exhibited significant potential in terms of reducing the cooling load. In contrast, climate zones that have cool nights, such as the arid Mediterranean, the warm Saharan Mediterranean, and the warm, semi-arid Mediterranean, have demonstrated a greater potential to benefit from nighttime natural ventilation.

Implementing window shading and natural ventilation together results in a cumulative amount of energy savings for cooling energy consumption. Although this cumulative effect is not equal to the numerical addition of the two technologies together, their application together showed a marked increase in energy savings compared to the application of either one alone (see Table 3).

Movable shading devices exhibited significant potential in terms of reducing the cooling demand in various climates. For example, in a semi-arid Mediterranean climate, it can reduce approximately 71% of the cooling consumption. Additionally, in hotter climates, such as the cool, arid Mediterranean zone or the hot and warm Saharan Mediterranean zones, energy savings of up to 48.4%, 23%, and 53% can be achieved, respectively. Nighttime natural ventilation provides a notable contribution in terms of passive cooling. Climate conditions have had a major impact on the effectiveness of nighttime natural ventilation; therefore, in climates characterized by cool summer nights, great benefits have been demonstrated, with savings of 12%, 11%, and 14% of the cooling energy demand achieved in the arid Mediterranean, warm Saharan Mediterranean, and warm, semi-arid Mediterranean climates, respectively. In warm night climates, such as the hot Saharan Mediterranean, the ability of natural ventilation to reduce energy consumption significantly decreases to savings of approximately 1.3% of the total cooling demand.

The significant effect of window shading and nighttime natural ventilation in reducing the cooling demand in different climates will be reflected directly in the inside of the building environment. Reducing the temperature inside the buildings through the use of natural means allows for a larger-scale move away from the use of high-cost, artificial cooling devices. This will improve the general health of the population and reduce the negative effects of the increase in heat and its associated diseases. Nighttime natural ventilation will contribute significantly to reducing the effect of the urban heat island phenomenon, which is associated with reduced thermal comfort and causes many dermatological diseases.

## 4. Conclusions

Building occupants play a significant role in energy consumption by interacting with their environment to reduce cooling loads and associated dermatological health distresses. In this study, we introduce two simple strategies, which are movable window shadings and nighttime natural ventilation, to achieve the goal of reducing energy consumption in residential buildings through passive cooling.

The application of these two approaches is controlled by building occupants. A traditional Jordanian building that adheres to all Jordanian energy codes was simulated and tested in four different climates. The effect of implementing the two strategies on cooling demand was investigated. The outcomes show that practicing those manageable methods can decrease the energy used for cooling, even in well-designed buildings that have sufficient properties, such as thermal insulation. Movable shading devices exhibit a significant potential to reduce the cooling demand in various climates. In a semi-arid Mediterranean climate, it can reduce cooling consumption by around 71%. Further, in hotter climates like the cool, arid Mediterranean zone, and the hot and warm Saharan Mediterranean zones, the potential energy savings can reach up to 48.4%, 23%, and 53%, respectively. Nighttime natural ventilation can provide a notable contribution in terms of passive cooling, and climate conditions have a major impact on the effectiveness of nighttime natural ventilation. Therefore, the climates that are characterized by cool summer nights show great energy benefits, reaching up to 12.25%, 11.15%, and 13.74% savings in cooling demand in the arid Mediterranean, warm Saharan Mediterranean, and warm, semi-arid Mediterranean zones, respectively. In warm night climates, such as the hot Saharan Mediterranean, the ability of nighttime natural ventilation to results in energy savings significantly decreased to around 1.3% savings relative to the total cooling demand.

The final results indicate that window shading has a significant potential to reduce the cooling load in different climate zones compared to natural ventilation, which offers high energy-saving abilities in climates that have cold nights, while its abilities in hot climates where the nights are moderate are limited. This knowledge of how the occupants sustain their thermal comfort without using air-conditioning systems could be implemented by raising their awareness on how to use their homes, such as by taking advantage of the summer breeze and the thermal mass, and preventing summer radiation from entering the house. Finally, these low-cost, passive design techniques, in addition to saving a significant amount of energy, will be key techniques in the prevention of some dermatological diseases. These techniques will be more effective than some medicines in relation to dermatological diseases, as “prevention is much better than cure”.

## Figures and Tables

**Table 1:** Case Study Building Features.

Model Parameter	Value
Roof (U-Value)	0.535 (W/m^2^.k)
External wall (U-Value)	0.563 (W/m^2^.k)
Internal wall (U-Value)	1.652 (W/m^2^.k)
Window type	double glass Generic BLUE
Window (U-Value)	0.505
Window-to-wall rate	30%
Infiltration rate	1.5 ACH
Cooling set point	24 °C

**Table 2:** Shading device properties.

Shading Type	Shading Position	Shade Properties			
		Conductivity (W/m.k)	Solar Transmittance	Solar Reflectance	Long-Wave Emissivity
Diffusing shades	outside	0.1	0.1	0.8	0.9

**Table 3:** Annual amounts and percentages of cooling energy savings from the use of different passive techniques in different climates.

	Semi-arid Mediterranean, Warm		Saharan Mediterranean, Hot		Saharan Mediterranean, Warm		Arid Mediterranean, Cool	
	Cooling Energy Savings (kWh/year)	Percentage of Cooling Energy (%)	Cooling Energy Savings (kWh/year)	Percentage of Cooling Energy (%)	Cooling Energy Savings (kWh/year)	Percentage of Cooling Energy (%)	Cooling Energy Savings (kWh/year)	Percentage of Cooling Energy (%)
**Window shading**	820	71.3	1759	23.3	1546	48.4	1910	52.7
**Nighttime natural ventilation**	158	1.6	119	1.6	356	11.2	444	12.3
**Window shading and nighttime natural ventilation**	877	76.2	1860	24.6	1712	53.6	2096	57.8

## Data Availability

This study did not report any data.
